# TNF-α promoter polymorphism: a factor contributing to the different immunological and clinical phenotypes in Japanese encephalitis

**DOI:** 10.1186/1471-2334-12-23

**Published:** 2012-01-26

**Authors:** Sujit Kumar Pujhari, Radha Kanta Ratho, Sudesh Prabhakar, Baijayantimala Mishra, Manish Modi

**Affiliations:** 1 Department of Virology, Postgraduate Institute of Medical Education and Research, Chandigarh 160012, India; 2 Department of Neurology, Postgraduate Institute of Medical Education and Research, Chandigarh 160012, India

## Abstract

**Background:**

More than three billion populations are living under the threat of Japanese encephalitis in South East Asian (SEA) countries including India. The pathogenesis of this disease is not clearly understood and is probably attributed to genomic variations in viral strains as well as the host genetic makeup. The present study is to determine the role of polymorphism of TNF-alpha promoter regions at positions -238G/A, -308G/A, -857C/T and -863C/A in the severity of Japanese encephalitis patients.

**Methods:**

Total of 142 patients including 66 encephalitis case (IgM/RT-PCR positive), 16 fever cases (IgM positive) without encephalitis and 60 apparently healthy individuals (IgG positive) were included in the study. Polymerase chain reaction restriction fragment length polymorphism (PCR-RFLP) using site specific restriction enzymes were implemented for polymorphism study of TNF alpha promoter.

**Results:**

Following the analysis of the digestion patterns of four polymorphic sites of the TNF- alpha promoter region, a significant association was observed between the allele -308A and -863C with the patients of Japanese encephalitis.

**Conclusions:**

TNF- alpha 308 G/A has been shown to be associated with elevated TNF- alpha transcriptional activity. On the other hand, polymorphism at position -863C/A in the promoter region has been reported to be associated with reduced TNF- alpha promoter activity and lower plasma TNF levels. As per the literature search, this is the first study to identify the role of TNF- alpha promoter in JE infection. Our results show that subjects with - 308A and -863C alleles are more vulnerable to the severe form of JE infection.

## Background

Japanese encephalitis (JE) is an important flaviviral disease of public health concern in Eastern and South-East Asian (SEA) countries. Approximately 50,000 cases are being reported annually to WHO with a case fatality of 10,000 cases [1]. On account of globalization and climatic change, there is a gradual spread of the virus into naïve geographical areas including Australia and UK, however, fortunately there is no report of local spread of JE in UK [2,3]. In the past, the neurotropic nature of Japanese encephalitis virus (JEV) often posed epidemic threats of acute encephalitic syndrome (AES) in SEA countries including the Indian subcontinent [4,5]. Commonly the spectrum of JEV infection ranges from an asymptomatic infection to meningoencephalomyelitis with cortical damage and cord lesions. An overt case of JE carries a poor prognosis with a mortality of 10-30%; one third of recovered cases land into neurological sequelae whereas complete recovery is expected in the remaining one third of patients [6].

Tumor necrosis factor is a multifunctional pro-inflammatory cytokine with a role in modulation of acute inflammation and host innate immunity. Its association towards disease progression has well been observed in several acute and chronic infections and autoimmune diseases. The reactivation of latent tuberculosis following treatment with anti-TNF antibody (Infliximab) has been reported [11], indicating TNF as a key cytokine towards the development of resistance to the tubercle bacilli and other intracellular pathogens. Even an excess production of TNF often proved to be detrimental in cerebral malaria, erythema nodosum leprosum and leishmaniasis [12-17].

The variations in the capacity to produce cytokines in different individuals have been attributed to the existence of polymorphisms within the regulatory regions or signal sequences of the cytokine genes. The present study aimed to associate the role of TNF-α promoter regions at positions -238, -308, -857 and -863 with disease progression and outcome in patients with Japanese encephalitis. The single nucleotide polymorphisms (SNPs) have been targeted because of their putative role on the promoter activity which might influence the TNF-α production and immunologic homeostasis.

## Methods

All the subjects (n: 142) included in the study were enrolled during the post monsoon period (August-December 2007-2009) i.e. JEV transmission season with informed consent. Of the total, 66 were JE patients [IgM (n: 56)/RT-PCR (n: 10) positive] with encephalitic features collected from inpatients departments of PGIMER, Chandigarh, 16 were JE IgM positive cases who presented with acute febrile illness and the rest (n: 60) were apparently healthy subjects previously exposed to JEV infection (confirmed by detection of JEV specific IgG antibody) collected from the rural areas endemic to Japanese encephalitis belonging to the neighboring rice growing states of Chandigarh. A clinical-case definition of JE was followed as per National Vector Borne Disease Control Programme, Govt. of India. Febrile illness of variable severity associated with neurological symptoms ranging from headache to meningitis or encephalitis were labeled as Acute Encephalitic Syndrome (AES). Symptoms can include headache, fever, meningeal sign, disorientation, coma, tremor, generalized paralysis, hypertonia and loss of coordination. Patients with suspected AES showing JE specific IgM antibody in the cerebro-spinal fluid (CSF) only/in paired samples of CSF and blood or JEV RNA in CSF [9,18] are labeled as confirmed JE cases whereas those with only JE IgM positivity in serum are considered as probable JE cases [19]. The possibility of cross-reactive antibodies to dengue virus was taken into account and all the samples (fever, encephalitis) were also screened for dengue specific IgM antibodies (Dengue specific IgM Ab ELISA; NIV, Pune, India) and were found to be nonreactive. From each subject, approximately 3 ml of blood was collected aseptically by trained health personnel for genomic DNA extraction (1 ml anti-coagulated blood) and viral serology (2 ml blood in sterile plain vial). The details of serology and viral genome detection procedures were described previously [9].

Genomic DNA was extracted as per the manufacturer's protocol using QIAGEN DNA extraction kit. For the detection of SNPs at TNF-α promoter region, gene specific PCR was performed followed by restriction fragment length polymorphism (PCR-RFLP). The positions -863C/A, -857C/T, -308G/A, and -238G/A were amplified using the published primer sequences and amplification profiles [20]. These PCR products were further subjected to restriction digestion with respective restriction enzymes as shown in Table 1 and the digested products were observed in ethidium bromide stained 3% agarose gel. The allelic type was determined according to the presence or absence of the digested product of the desired length. Adequate precautionary measures were taken for cross contamination during PCR amplification; RFLP and 50% of the samples were repeated to maintain the quality of the data.

**Table 1:** Restriction enzymes and digestion patterns for genotyping of TNF promoters

Promoter position	Restioction enzymes	Digestion patterns	Genotypes
-238 G/A	MspI	132,20	GG

		152,132, 20	GA

		152	GG

-308 G/A	StyI	123,20	GG

		143,123,20	GA

		143	AA

-857 C/T	HincII	106 25	CC

		131,106 25	CT

		131	TT

-863 C/A	StyI	108, 25	CC

		133,108, 25	CA

		133	AA

### Statistical analysis

The data was evaluated by chi-square (*χ*^2^) analysis using standard statistical software with a P value of < 0.05 being statistically significant. Genotypic frequencies were examined by Hardy-Weinberg equilibrium test. EPI version 6.2 was implemented for estimation of the *χ*^2 ^value, odds ratios and 95% confidence intervals for genotypic and allelic frequencies.

## Results

### Demographic characteristics of the subjects

The male:female ratio in the encephalitis, fever and control groups were 55/11, 10/6 and 19/41 respectively. It was interesting to note that children below 16 years age group were more affected and males outnumbered the females in the group of patients with encephalitic features (Table 2).

**Table 2:** Subject details

	Encephalitis (n = 66)	Fever (n = 16)	Control (60)	Χ^2 ^P Value
Age (Year mean ± SD)	16 ± 12.543	30.76 ± 14.942	21.72 ± 10.187	23.902 .001**

Sex(Male/Female)	55/11	10/6	19/41	29.148 .001**

Male/Female in %	83.3/16.7	62.5/37.5	31.7/68.3	

### Genotypic distribution and allelic frequency

The genotypic and allelic frequencies of -863C/A, -857C/T, -308G/A, and -238G/A were calculated (Table 3). Allele and genotypic frequencies were in Hardy-Weinberg equilibrium. The frequency distribution of the homozygous GG and heterozygous GA at -308 in encephalitis group was statistically significant when compared with fever (GG: *Χ*^2 ^= 15.96, p = 0.001**, OR = 11.56, 95% CI = 2.61-58.51; GA: *Χ*^2 ^= 15.96, *p *= 0.001**, OR = 0.09, 95% CI = 0.02-0.38) and control group (GG: *Χ*^2 ^= 42.28, *p *= 0.001**, OR = 15.11, 95% CI = 5.73-41.35; GA: *Χ*^2 ^= 42.28, *p *= 0.001**, OR = 00.07, 95% CI = 0.02-0.17). Again the distribution of the homozygous CC and heterozygous CA genotypes at -863 in encephalitis groups were statistically significant as compared with control group (CC: *Χ*^2 ^= 23.51, *p *= 0.001**, OR = 0.12, 95% CI = 0.04-0.33; CA: *Χ*^2 ^= 11.69, *p *= 0.001**, OR = 9.74, 95% CI = 1.96-65.38) (Table 4). Out of the 66 encephalitis patients, 9 had fatal outcome, of which 7 had the typical allelic distribution of -308A and -863 C genotypes.

**Table 3:** Genotypic and allelic frequencies of TNF-α polymorphism

		Control	Encephalitis	Fever
**-**238	GA	4(6.7%)	6(9.1%)	2(12.5%)

	GG	56(93.3%)	60(90.9%)	14(87.5%)

	G	96.67	95.45	93.75

	A	3.33	4.5	6.25

**-308**	GA	9(15.0%)	48(72.7%)	3(18.75%)

	GG	51(85.0%)	18(27.3%)	13(81.25%)

	G	92.5	36.37	90.62

	**A**	**7.5**	**63.63**	**9.38**

**-**857	CC	41(68.3%)	50(75.8%)	12(75%)

	CT	19(31.7%)	10(15.2%)	4(25%)

	TT	0	6(9.1%)	0

	C	84.16	83.33	87.5

	T	15.84	16.67	12.5

**-863**	AA	16(26.7%)	5(7.6%)	1(6.25%)

	CA	14(23.3%)	2(3.0%)	0

	CC	30(50.0%)	59(89.4%)	15(93.75%)

	A	38.33	9.09	96.87

	**C**	**61.67**	**90.91**	**3.13**

**Table 4:** Statistical comparison of Genotypic and allelic frequencies of TNF-α polymorphism between different groups

		Control Vs Encephalitis				Control Vs Fever				Fever Vs Encephalitis			
**Loci**	**Genotype**	**Χ^2 ^Value**	**P-Value**	**OR**	**95% CI**	**Χ^2 ^Value**	**P-Value**	**OR**	**95% CI**	**Χ^2 ^Value**	**P-Value**	**OR**	**95% CI**

-238	GA	0.25	0.43	0.71	0.16-3.06	0.59	0.44	0.5	0.07-4.42	0.17	0.48	1.43	0.18-9.28

	GG	0.25	0.43	1.4	0.33-6.29	0.59	0.44	2	0.23-14.90	0.17	0.48	0.7	0.11-5.64

-308	GA	42.28	0.001**	00.07	0.02-0.17	0.13	0.48	0.76	0.15-4.17	15.96	0.001**	0.09	0.02-0.38

	GG	42.28	0.001**	15.11	5.73-41.35	0.13	0.48	1.31	0.24-6.47	15.96	0.001**	11.56	2.61-58.51

-857	CC	0.86	0.35	0.69	0.29-1.62	0.27	0.42	0.72	0.17-2.87	0.001	0.58	0.96	0.24-4.13

	CT	4.84	0.02	2.60	1.01-6.76	0.27	0.42	1.39	0.35-5.39	0.88	0.27	1.87	0.41-8.13

	TT	6.93	0.01			3.03	0.07	5.45	0.65-119.49	26.7	0.001		

-863	AA	8.25	0.04	4.44	1.38-15.09	4.56	0.03			0.03	0.66	0.81	

	CA	11.69	0.001**	9.74	1.96-65.38	10.01	0.001**	0.07	0.00-0.54	0.50	0.64		

	CC	23.51	0.001**	0.12	0.04-0.33	0.59	0.44	0.5	0.07-4.42	0.28	0.51	1.78	0.19-41.45

## Discussion

The human and murine model studies revealed that the outcome in infectious diseases is influenced by TNF-α. In meningococcal meningitis patients, low TNF-α production is associated with tenfold increase in mortality [21] whereas the severity of the disease is directly proportional to the TNF-α levels in CSF and blood of JE patients [10].

The patho-physiology of the CNS entry by neurotropic viruses is still an enigma. The role of inflammatory cytokines has been amply emphasized towards disrupting the integrity of the blood brain barrier in West Nile Virus (WNV) infections. TLR-3 mediated expression of TNF-α leads to loss of integrity of the endothelial cells in the brain, possibly by mediating the passage of viral particles and virus loaded immune cells into the CNS [22,23].

Following JE infection, approximately 1 in 300-1,000 persons would turn out with acute encephalitis and majority abort the infection sub clinically. The present study was aimed at the host factors governing pathogenesis of JE. There is no correlation between the disease severity and the circulating JE genotypes. However, a possible correlation is observed between the severity of JE and the level of JEV specific IgM antibody in the serum and CSF [10].

**Figure 1:**
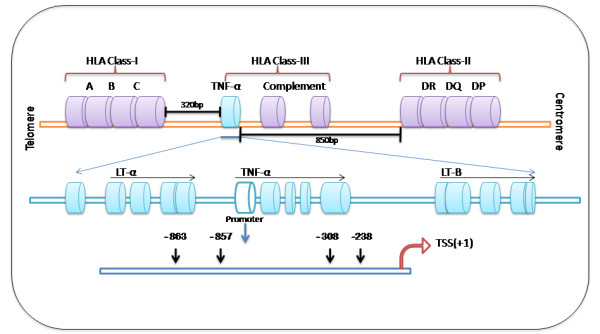
**Map of TNF-α promoter and TNF-&#945 gene on chromosome 6. **Animal studies have pointed towards the association of particular single and/or multiple nucleotide changes in the viral genome with altered virulence but the degree of severity and disease outcome in JE do not correlate with virus genotypes [7-9]. Thus, the concept of the host genetic makeup towards disease modulation has been postulated. Elevated levels of tumor necrosis factor alpha (TNF-α), a central mediator of immune response, in both serum and CSF samples in patients with JE have been associated with poor prognosis [10].

The TNF-α gene is located within the class III region of the major histocompatibility complex (MHC) between HLA-B and DR, (Figure 1) and its expression in the host is controlled in a regulated manner at the transcriptional as well as post transcriptional level. Polymorphism of TNF-α promoter region has been shown to play a role in the development of autoimmune diseases and several infections like malaria, leishmaniasis, hepatitis B virus, hepatitis C virus, human papiloma virus etc. [13,15,17,24].

The -308A allele in the TNF-α promoter (also known as TNF2) is a G/A transition and is the most studied SNP in this gene. In case of HBV infection, the presence of -308A and -863C was associated with viral clearance [15], whereas SNP -238G has been shown to be associated with protection against chronic hepatitis in the Chinese and German population [14,16]. In hepatitis C virus infection, the haplotype -863C/-308G was associated with viral persistence in African Americans [12]. Similarly the existence of -308A SNP in pediatric population positively correlated with the severity of dengue viral infection in children.

Single nucleotide polymorphisms (SNPs) in the TNF-α promoter region at positions -863C/A, -857C/T, -308G/A and -238G/A with reference to the transcription start site have been analyzed based upon the TNF secretion capacity. TNF-α -308G/A has been shown to be associated with elevated TNF-α transcriptional activity. On the other hand, polymorphism at position -863C/A in the promoter region has been reported to be associated with reduced TNF-α promoter activity and lower plasma TNF levels. As per the literature search, this is the first study to identify the role of TNF-α promoter in JE infection. Our results show that subjects with -308A and -863C alleles are more vulnerable to the severe form of JE infection. Logistic regression could not be performed in this study due to limited numbers of events which happens to be one of the limitations and warrants caution with regards to the conclusion.

A combined effect of prolonged exposure to high level of pro-inflammatory cytokines (TNF-α) combined with reduced release of anti-inflammatory cytokines IL-10 and IL-4 in encephalitis conditions may cause immunologic imbalance landed with poor prognosis [25]. Host genetic display of cytokine genes and their regulatory sequence alters the expression patterns of the different cytokines. In summary, the present study supports the potential role of genetic variation in the TNF-α promoter region in susceptibility to JEV infection.

## Conclusion

Subjects with -308A and -863C alleles were more vulnerable to the severe form of JE infection and of the total 9 deaths, 7 had -308A and -863C allelic distribution. At the verge of entering into the era of personalized medicine, understanding the association of mutations with infectious disease is very important. This information will help the physician to become aware of a prior possible outcome of the patient and thus to follow an appropriate management protocol for the patients.

## Competing interests

The authors declare that they have no competing interests.

## Authors' contributions

SKP, RRK, SP, BMM and MM collectively designed the project work. SKP has done the sample collection and experimental work. SP and MM are the clinicians of this study. SKP, RRK, and BMM analyzed the data. All the authors contributed in manuscript writing. All authors read and approved the final manuscript.

## Pre-publication history

The pre-publication history for this paper can be accessed here:

http://www.biomedcentral.com/1471-2334/12/23/prepub
